# Is Lifelong Knee Joint Force from Work, Home, and Sport Related to Knee Osteoarthritis?

**DOI:** 10.1155/2012/584193

**Published:** 2012-07-17

**Authors:** Charles R. Ratzlaff, Mieke Koehoorn, Jolanda Cibere, Jacek A. Kopec

**Affiliations:** ^1^Division of Rheumatology, Immunology, and Allergy, Brigham and Women's Hospital, 75 Francis Street PBB3, Boston, MA 02115, USA; ^2^School of Population and Public Health, University of British Columbia, 5804 Fairview Avenue, Mather Building, Vancouver, BC, Canada V6T 1Z3; ^3^Arthritis Research Centre of Canada, 895 West 10th Avenue, Vancouver, BC, Canada V5Z 1L7; ^4^Division of Rheumatology, University of British Columbia and Gordon & Leslie Diamond Health Centre, 10th Floor, 2775 Laurel Street, Vancouver, BC, Canada V5Z 1M9

## Abstract

*Purpose*. To investigate the association of cumulative lifetime knee joint force on the risk of self-reported medically-diagnosed knee osteoarthritis (OA). *Methods*. Exposure data on lifetime physical activity type (occupational, household, sport/recreation) and dose (frequency, intensity, duration) were collected from 4,269 Canadian men and women as part of the Physical Activity and Joint Heath cohort study. Subjects were ranked in terms of the “cumulative peak force index”, a measure of lifetime mechanical knee force. Multivariable logistic regression was conducted to obtain adjusted effects for mean lifetime knee force on the risk of knee OA. *Results*. High levels of total lifetime, occupational and household-related force were associated with an increased in risk of OA, with odds ratio's ranging from approximately 1.3 to 2. Joint injury, high BMI and older age were related to risk of knee OA, consistent with previous studies. *Conclusions*. A newly developed measure of lifetime mechanical knee force from physical activity was employed to estimate the risk of self-reported, medically-diagnosed knee OA. While there are limitations, this paper suggests that high levels of total lifetime force (all domains combined), and occupational force in men and household force in women were risk factors for knee OA.

## 1. Introduction

The promotion of physical activity (PA) is a major public health initiative in many countries due to its protective effect on numerous major health problems [1], including Canada and the US where public health bodies recommend 30 to 60 minutes of moderate-to-vigorous activities per day. However, there has long been a concern that such promotion could lead to a rise in hip and knee OA, the major public health problem in musculoskeletal medicine and a leading cause of chronic disability [2]. While there is a broad agreement that PA is an important determinant of joint health, it is unclear what amount and type of PA are beneficial or pose a risk. In short, despite numerous studies, the association between PA and joint health is complex and poorly understood.

While different study designs, case definitions, sampling frames, and size play a role, the wide variation in how PA is defined is the most probable reason for the uncertainty. There is a lack of valid, reliable, and standardized instruments across studies, substantial measurement error, variation in the period and nature of PA measured, and failure to measure the most relevant aspect of PA-joint load [3]. Where accurate and precise measures are available, they are impractical for use in population-based research. The goals of this study were to address these gaps by (1) measuring historic PA, a key variable given the long latency and asymptomatic induction period of OA that, while potentially imprecise, is likely more important than accurate measure of an irrelevant variable such as current activity, (2) examining loads applied to the joint using quantitative joint loading units that allowed for assessment of cumulative force from PA, including dose response, and (3) including household activity (in addition to occupation and sport/recreation) to capture all three primary domains of activity.

The purpose of this study was to evaluate the association between levels of lifetime knee joint force and knee OA in a large Canadian sample of community dwelling adults.

## 2. Methods

**Data Source and Study Population**

The source population was community-dwelling members of the Canadian Association of Retired Persons, Canada's largest 50-plus advocacy group with 350,000 members. Data collection methods have been previously described [4, 5]. Briefly, recruitment was via direct email to 28,000 members and to 100,000 additional members via an advertisement in an online newsletter, appearing in two consecutive newsletters. All e-mails and newsletters contained hyperlinks or banner advertisements directing subjects to the study website. Through these methods, subjects across Canada were recruited over the Internet. Incentives included $1,500 in lottery prizes. After completing an electronic consent form, subjects were given password access to the questionnaire. All data collection was web-based and used skip logic technology that allowed subjects to follow individualized paths through the survey, moving forward based on responses to previous questions. Extensive pre- and pilot-testing was carried out to ascertain best recruitment methods [6], survey duration, navigation, and to ensure that respondents could understand items, retrieve information, and make appropriate estimations. A secure website for the study allowed subjects to save responses and return later. The baseline questionnaire, carried out from June to September 2005, took 60 to 90 minutes to be completed.

**Figure 1:**
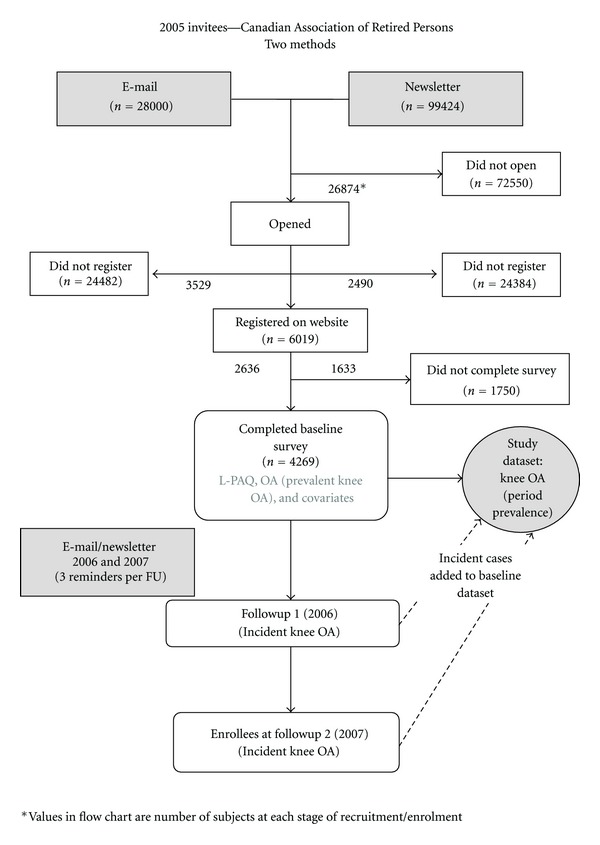
Lifetime Physical Activity and Joint Health Study: recruitment and enrolment.

Response rates have been previously described [4] and are presented in Figure 1. Individuals who completed the baseline survey were contacted by email and letter for follow-up surveys at approximately one (May 2006) and two years (June 2007). Follow-up surveys inquired about knee joint health using the same questions as the baseline survey.

### 2.1. Design

A period prevalence study using a cross-sectional design was utilized, as there were an insufficient number of incident knee OA cases to power the study to meet the objectives. To compile the dataset, subjects reporting OA at any of the three time points (Figure 1) were identified as cases. Baseline exposure data was used. While the inclusion of prevalent cases could potentially limit the ability to delineate cause and effect, the approach was justified on several grounds, and steps were taken to guard against reverse causality. First, increasing the number of cases by combining prevalent and incident cases allowed for greater study power to assess gender-specific relationships between PA and OA while including a number of covariates, and assessing dose-response relationship of PA with OA. Second, a new measure of exposure—quantitative lifelong joint force from work, sport, and household activity measured in joint loading units—was investigated for the first time. Third, the relationship between the historic PA measured in this study (up to age 50, as discussed in Methods) and the time of diagnosis for the vast majority of cases is separated in time, providing some protection against reverse causality. In addition, the highest levels of lifetime PA for most people occur prior to the age of 50 [5]. Lastly, since OA is not a curable condition, prevalence is a relatively stable reflection of disease frequency.

### 2.2. Physical Activity Measurement

Lifetime physical activity was assessed using the Lifetime Physical Activity Questionnaire (L-PAQ), whose development and validation has been described previously [7]. In summary, the L-PAQ is a self-administered, web-based questionnaire that was based on existing instruments [8–10], developed using the principles of construct validation, adapted for self-administration over the Internet, incorporated skip logic technology, and expanded to capture more detailed information including bodily movements involving the knee. Using a subsample of the current study, intraclass coefficients for reliability ranged from 0.65 to 0.89; convergent validity testing against two validated lifetime questionnaires resulted in Spearman correlation coefficients ranged from 0.41 to 0.71 [7].

The LPAQ measures lifetime PA across three domains: sport/recreation, occupation, and household and had been described previously [4, 5]. Sample questions in each domain are shown in Appendix A. Briefly, in the sports/recreational section, respondents were provided with a list of 64 possible sports and were permitted to add other sports. Data collected included the duration, frequency, and average length of time per occasion, and time spent per hour (none, 1–5, 5–15, 15–30, 30–45, and 45–60 minutes per hour) in eight bodily positions or movements. The occupational section used an open format in which respondents, for each occupation ever held, provided details on job title or type, duration, average hours per week, and whether the job was full time, part time, or seasonal and time spent per eight-hour segment in nine bodily positions or movements. Household activity covered four areas: (1) caring for children; (2) caring for elderly or disabled individuals; (3) gardening; (4) housework. For each household area of activity, participants were prompted to provide detailed information on duration and average number of hours per week of participation and were required to report time spent in an 8-hour period for each of the bodily movements as per the occupational section (Appendix B—list of activities and assigned joint force).

### 2.3. Cumulative Peak Force Index (Bodyweight-Hours)

To obtain a measure of cumulative joint force at the knee, a cumulative peak force index (CPFI) score was estimated at the knee and has been described previously [5] as the product of time spent in a specific activity (total lifetime hours), bodyweight (BW), and typical peak joint force for that activity (%BW), (i.e., CPFI score (bodyweight-hours) = total lifetime hours ∗ bodyweight ∗ typical peak joint force, per each activity).

As previously described [4, 5], the CPFI is a newly proposed measure, and steps were taken to validate and/or ensure the greatest precision in the components that comprise it. In addition to validation of the L-PAQ [7], self-reported bodyweight and height were utilized, measures with established validity properties [11] used in numerous epidemiologic studies. We improved on a single self-report of bodyweight by asking about it at three time points (baseline survey, age 20, maximum lifetime), and deriving a lifetime bodyweight trajectory, interpolated using a Lowess (nonparametric smooth) curve. The third component of the measure was the typical peak joint force assigned to each activity (Appendix B). These values were determined after an extensive literature [12–40] (full bibliography available on request) that prioritized *in vivo* studies and incorporated judgments about data quality and study rigor. Data were synthesized and a consensus achieved by a panel of experts from biomechanical engineering, rheumatology, physiotherapy, and musculoskeletal epidemiology.

The force exposure variable was operationalized as exposure prior to the age of 50. The main reason for this was to capture the primary PA exposure prior to the diagnosis of OA (and first symptoms) for the vast majority of cases, and to minimize the effects of subclinical, undiagnosed, or early OA on PA patterns. Support for this approach was also drawn from the previous study on lifetime trajectories [5] that revealed that peak exposure window for PA is prior to 50, being at its highest lifetime levels from ages 30–45.

### 2.4. Case Ascertainment

An algorithm was used to ascertain knee OA cases, requiring that subjects report both “health-professional-diagnosed knee OA” and “pain, aching, or stiffness on most days” at any of the 3 time points (baseline, 1st and 2nd followups). The questionnaire item on health-professional-diagnosed OA informed subjects that OA was distinct from other MSK diseases (e.g., rheumatoid arthritis and osteoporosis), and a response confirmation to this question was required in a follow-up item.

The reliability and validity of self-reported knee OA was carried out in a substudy and has been previously published [41]. Briefly, using the American College of Rheumatology (ACR) clinical classification criteria for knee OA [42] as a diagnostic referent, sensitivity was 0.76, specificity 0.98, positive predictive value 0.87, and negative predictive value 0.95. Kappa value was 0.76 indicating substantial agreement beyond chance [43]. Our findings are consistent with other studies comparing self-report medically diagnosed OA and clinical OA [44–46].

### 2.5. Covariates

The baseline questionnaire measured known knee health risk factors and included gender, age, body mass index (BMI), ethnicity (Asian, Black, Caucasian, First Nations, Hispanic, and others), and education (elementary, high school, postsecondary, and trade/technical).

### 2.6. Injury

The following question from the baseline questionnaire was used to determine the presence of significant knee injury: “Have you ever had a knee injury that required you to use a walking aid (e.g., cane or crutch) for at least one week?”   Follow-up questions included the age at injury (if more than one injury, the time of first injury was requested). Only injuries that occurred before the diagnosis of OA were included in analysis.

### 2.7. Statistical Methods

The prevalence of knee OA and covariates for the study sample were calculated. CPFI values for each activity were summed for sport, occupation, and household domains for each 5-year period of a person's lifetime to factor in changes in bodyweight over time, and these domain values were then summed to give a total CPFI value [5]. For the total force (CPFI) variable, subjects were categorized into quintiles of exposure for the overall distribution, prior to stratification by gender. For the domain specific analysis, the quintiles were based on the relative distribution within each domain (occupation, household, and sport), again prior to stratification by gender (e.g., quintile 5 for occupation was at the same joint loading level for both sexes). Crude odds ratios were calculated for the relationships between knee OA and joint force variables and other study covariates. Potential collinearity and interaction between covariates was examined on a bivariate basis.

Covariates were selected based on scientific knowledge and the conceptual framework of causal pathways to knee OA. Factors associated with an increased risk of knee OA, which also could be confounders, such as age, previous injury, and BMI were adjusted for in all the analyses. The potential effect of one domain on another (e.g., occupational activity when assessing the sport-OA relationship) was also potentially confounding and included. Men and women were examined separately because of known gender differences in disease prevalence, physical activity profiles, injury rates, and BMI. Reference categories were the lowest CPFI, youngest age tertile (<58), normal BMI (20.0–24.9), and no previous injury. Test for trends was obtained by treating the categories (quintiles) of the CPFI variable as continuous and testing the slope for significance; the models contained all relevant adjustment covariates.

Multiple logistic regression was used to examine if levels of total knee CPFI were associated with a risk of knee OA, controlling for age, previous injury, and BMI. An additional adjusted analysis was carried out investigating the separate effect of occupational, sport, and household CPFI (adjusted for the other domains). Analyses were performed using SPSS version 18 (Chicago, IL, USA).

## 3. Results

Subject characteristics for the sample are presented in Table 1. Frequencies of outcome, exposure, and covariates for this study are provided in Tables 2 and 3. The prevalence of knee OA was 22.4% for the sample overall—17.8% for men and 25.1% for women (Table 2). Twenty-six percent of the sample were of normal BMI, with approximately 72% being either overweight (39.9%) or obese (31.5%). More men than women (47.7 to 36.1%) were overweight, and more women than men were obese (33.4 to 29.5%). Twenty percent had a history of previous knee joint injury (24% in men and 18% in women).

The prevalence of subjects in occupational and household quintiles varied substantially by gender (Table 3). For example, in the male household strata, the largest prevalence (36.3%) was in the lowest (referent) quintile of exposure, while the smallest prevalence (6.2%) was in the highest quintile of exposure. For women, the largest prevalence (27.5%) was in the highest quintile of exposure and the smallest prevalence in the referent quintile (11.1%). For occupational force, these relative proportions were reversed by gender, though the percentages were slightly different (Table 3).

Crude ORs provided evidence that older age, previous knee injury, obesity in men, and overweight and obesity in women were associated with knee OA (Table 2). Compared with the lowest category of total lifetime knee force the highest category (5th quintile), in men and 3rd, 4th, and 5th quintiles in women were crudely related to knee OA (Table 2). For domain-specific lifetime force, the 5th quintile of occupational force in men and both occupational and household activity in and women were crudely related to OA (Table 3).

Adjusted ORs for risk factors on knee OA obtained from multiple logistic regression are presented in Tables 2 and 3. The strength of association for knee OA and total lifetime force remained significant for the highest force quintile in both men (1.70; 95% CI—1.06, 2.70) and women (1.52; 95% CI—1.15, 2.02). In domain-adjusted models, occupational force in men and household force in women were related to knee OA and tests for trend in these domains, as well as in total lifetime force (both men and women), were significant (*P* < 0.001).

Sport/recreational force was not related to knee OA. After adjustment, older age, injury, and high BMI remained significantly related to knee OA. Being overweight (BMI 25–29.9) increased the risk of knee OA for women (OR 1.69; 95% CI—1.31, 2.19) but was not related to knee OA in men. Obesity (BMI > 30) was a greater than two-fold risk for men and three-fold risk for women.

## 4. Discussion

This prevalence study on a large sample of Canadian adults presents a newly proposed measure of lifetime mechanical knee joint force based on hours in PA, bodyweight, and typical knee joint force for specific activities and relates it to self-reported knee OA. We provide evidence that while lifelong PA may generally be safe for the knee joint, that very high force from lifelong total force, and from high levels of total occupational force (men and women) and household activity (women), is a potential risk for knee OA. The results held after adjustment for known risk factors. These novel findings require confirmation in other populations and in longitudinal studies. Our results are consistent with previous studies that show overweight/obesity, age, female sex, and previous injury are significant risk factors for knee OA [47–55].

The results of this study must be compared cautiously with previous studies due to its cross-sectional design, how subjects were assembled, and how exposures and outcomes were measured. For example, no other studies evaluating PA and knee OA have used the Internet for data collection or have completely classified PA in terms of a joint loading variable over the long term, perhaps partly explaining inconsistent results from these past studies [44, 56–61]. Of note, the cross-sectional design may have resulted in reverse causality potentially attenuating risk estimates. Conversely, subjects with OA may have overreported prior PA exposure because they perceive that PA caused their OA (recall bias), potentially increasing the risk estimates [56]. Since this type of bias threatens most prevalence studies, emphasis should be placed on recent high-quality cohort studies evaluating the association between PA and OA.

A recent prospective cohort study with a 22-year-followup using physician-diagnosed OA, reported an adjusted OR for the heaviest category of physical demands at work compared with the lightest category of 18.3 for knee OA [62]. Verweij et al. [63], during 12 years of followup, recently reported that 463 of 1678 respondents (28%) developed clinical knee OA, and that a high mechanical strain score was associated with an increased risk of knee OA (HR 1.43, 95% CI 1.15–1.77) after adjustment for a number of covariates. Wang et al. [64], in a prospective cohort study of approximately 40,000 Australians with an average 5 years of followup, reported a composite sport and occupational exposure (past 6 months, measured at baseline) and found a risk for total knee joint arthroplasty for the vigorous level of activity (HR 1.42, 95% CI 1.08–1.86). Several studies from the Framingham cohort suggest that job activities may cause as much as 15% to 30% of knee OA in men [65, 66]. Felson et al. [65] reported that elderly persons (average age 70) in the highest quartile of PA at a baseline examination had over three times the risk of developing radiographic knee OA nine years later, when compared with those in the lowest quartile. McAlindon et al. [67] using longitudinal Framingham data reported that the number of hours per day of heavy physical activity was associated with the risk of incident radiographic knee OA (OR = 7.0 for 4+ hours heavy physical activity/day). No effects were observed from moderate and light PA. In contrast, a study by Hannan et al. [68] in the same cohort found no increase in the risk of knee OA with increasing physical activity. In the highest quartile of PA compared to the least active, the OR was 1.3 for men and 1.1 for women (both nonsignificant). Hart et al.[69], using data from the Chingford study, followed 715 women (mean age: 54 years) for 4 years with no radiographic knee OA at baseline and included the PA categories of walking, occupation, and sport/recreation. They found no relationship between incident knee OA and PA, while walking protected against joint space narrowing (OR = 0.4, 95% CI 0.2–0.9).

It is evident from these often-cited reports that, despite the longitudinal cohort designs, large samples and lengthy followups, and estimates for the risk of PA on knee OA vary extensively. While differences in eligibility criteria, covariates included in multivariable models and small samples may account for some of the disparity, the most likely reason is the wide variation in PA exposure measurement. Of note, most studies have not measured the joint-force aspects of PA nor attempted to completely classify PA (including historic PA) from all three major activity domains. Apart from the Verweij et al's study [63], none of the above studies considered PA from all three major activity domains or attempted to estimate the effect of activities in terms of joint force. The main finding of Verweij et al. was an OR of 1.43 (95% CI 1.15–1.77) for a high knee mechanical strain score, close to our reported OR's from the highest quintiles of total knee force, occupational force, and household force in women. Results are not directly comparable since apart from differences in design, the mechanical strain score was a ranking (1 to 4) of certain physical activities over the past 2 weeks (taken at baseline) and did not look at sex-specific differences in occupational and household activity. Consistent with the recent longitudinal cohort study of Toivanen et al. [62] and a number of longitudinal and case control studies [55–57, 68–75], we did not find a relationship between sport/recreational activity and knee OA. Studies that have shown a relationship between sport and knee OA have generally been in populations of athletes in specific sports with high knee forces [54, 76–81] and not from population-based studies, or the association has been explained by joint injury [76]. In population-based studies of lifetime activity, the highest sport/recreation rates typically occurs at a relatively young age, as it did in the current cohort, prior to the age of 25 [5]. Thus, high forces from sport later in life, when the joint may be more vulnerable, were not well-represented in this sample and may contribute to the lack of association here.

The prevalence of knee OA in our study was 22.4%, 17.8% in men and 25.1% in women. These gender differences in prevalence are consistent with previous large population-based North American studies for this age group [82–84]. Even though there is probably some misclassification, our definition, which required a medical diagnosis and the presence of pain on most days, is important since pain is usually the most important aspect of disease to patients and may precede X-ray change, potentially capturing earlier disease. We report the results of a validity study in a subsample of the current study comparing self-reported OA to clinical OA [41].

It was important to measure and simultaneously adjust for PA-related force from all three major activity domains. Most previous PA-OA studies have investigated one or two domains (usually sport and/or occupation). Given the high levels of household and occupational PA reported in our previous paper [5], omitting one or both of these domains leaves these studies vulnerable to confounding from the unmeasured domain(s).

 In studying all three domains separately by sex, we also observed relationships of PA with very high occupational force in men and household force in women. Questionnaires used in many previous studies did not assess the frequency, duration, and intensity of PA actually performed by women [85]. The majority of women's exposure to PA, particularly in older cohorts such as the current one, is due to accumulation of regular household activities [85–88]. While household activity may generally not be considered vigorous from an energy expenditure perspective and is often ignored in epidemiologic study of OA, there are many repetitive motions (e.g., stair climbing, squatting, and kneeling) and activities (e.g., gardening, lifting, and carrying) that are associated with high knee joint forces [20, 21, 28, 36] but have low energy expenditure.

This is only the second study to measure lifelong household load at the knee joint and relate it to knee OA, and the first to quantify household knee joint force from historic activity for the assessment of dose response. In the previous study by Sandmark et al. [89], exposure to physically demanding tasks at home was significantly associated with knee OA among women (but not men) and was the strongest risk factor for women among the physical load variables that were investigated in that study. Given that women have been shown to have higher PA than men when including household together with occupational and sporting activities [5], and that reasons for the higher prevalence of knee OA in women are not clearly elucidated [51], our findings provide preliminary evidence that the role of historic household PA requires further investigation. The role of occupational activity has received much more study, and is better understood in men than in women, in part because previous studies were based historically on male-dominated workforce cohorts [51]. Even though women often spend forty or more hours a week at a full-time job and from twenty to forty-five hours per week working in the home, questionnaires used in many previous studies do not assess the frequency, duration, and intensity of PA actually performed by women [85]. It has been shown that, when the definition of regular physical activity measured in surveys is expanded to include household activity, PA levels rise and associations with health outcomes are more evident—including protective relationships with cardiovascular disease and myocardial infarction [90], cancer [91], and an inverse relationship with all-cause mortality [85, 90]. Our finding of an increased risk of knee OA for the top quintile of occupational joint force is generally consistent with previous studies [62, 64] and several systematic reviews [92–94].

The CPFI, a quantitative joint force measure, together with a large sample allowed for evaluation of a dose-response relationship between lifelong force and knee OA. In the models where a significant relationship with knee OA was found (total force, occupation, and household), there was an increasing, significant trend in the ORs from lower to higher levels of CPFI, though only the ORs for the highest (5th) quintile reached statistical significance. While this requires confirmation and further delineation in future studies, the presence of dose response strengthens evidence for a causal relationship [95].

This study had several strengths, including a large sample drawn from the population and a sufficient number of cases to adjust for a number of covariates, the separate analyses of men and women (equivalent to including interactions with gender for all variables), and assessment of dose-response. Another strength was the use of detailed information on the duration, frequency, and joint loading aspects of historic activities, from all three main physical activity domains, allowing for relatively complete classification of the total volume of PA. Historic PA is a potentially important exposure in OA etiology given the lengthy induction and asymptotic latency period. Measuring current or recent levels of PA does not capture long-term joint forces, may miss etiologically important periods of exposure and is a poor proxy for cumulative lifetime exposure [96]. Lastly, many studies have used advanced disease markers, such as total joint arthroplasty or moderate-to-marked radiographic change as the outcome in assessing the role of PA. It is not clear whether the relationship with PA for early, symptomatic cases is the same as that observed for advanced or radiographically defined OA. Our cases definition allowed us to capture information on earlier stages of disease. This may be important in understanding modifiable risk factors that could play a role in a prevention strategy for OA, something not currently available.

There are a number of limitations that are important in interpreting the results of this study. Self-report of knee OA may lead to misclassification. In our examination of the measurement properties of our case definition [41], we noted that specificity was very high. This is critical for studies of risk factors, since low specificity (inclusion of many false positives among cases) causes a greater attenuation of effect than low sensitivity. PPV was also high, another important measure indicating that the vast majority of the cases identified in the survey were true cases. Nevertheless, we did not use radiography as part of the classification criteria for knee OA. Radiographic OA in the presence of symptoms is thought to represent the best definition of OA. However, X-ray change is associated largely with moderate-to-advanced disease [97], and there is only moderate agreement between pain and symptoms and X-ray changes [98]. Wu et al., in a study using a validated outcome instrument for knee OA based on arthroscopic visualization, suggest that the ACR clinical classification criteria can be used to identify patients with early articular cartilage loss, before any radiographic changes are evident [97]. However, it is probable that the false positives include not only subjects with early OA not captured by the ACR criteria [97], but also other causes of knee symptoms.

The results from this study may not be broadly generalizable. The subjects were fairly well-educated, predominantly Caucasian Canadians with access to public health care and Internet users. This method of data collection may not be as effective in low-income populations, and those with decreased access to medical care—important since we asked about medically-diagnosed osteoarthritis as part of the case definition. Further, since recruitment and enrolment of subjects was via the Internet, subjects were largely self-selected. Self-selection implies that the nature of the bias cannot be known with certainty [99]. Studies of subjects who participate in online research reveal that they are more likely to be older, females and have higher socioeconomic status [100]. Also, response rates for online recruitment and enrolment vary from traditional rates. Many more individuals potentially view invitations to participate in research, with most declining to participate, making validity of results more challenging to interpret. In online surveys, there is no single response rate-multiple metrics for calculating a response rate have been defined such as the participation rate and completion rate [101]. However, the goal of this study was not to describe characteristics of the population at large, but to assemble subjects to test hypotheses about PA-related knee joint force and knee OA in a large sample of individuals who met criteria for a disease and those who did not, sampled in the same way (internal validity).

The limitations of our measure of self-report of PA measures and construction of the CPFI variable have been discussed elsewhere [4, 5]. In short, self-reported PA measures require cautious interpretation because of large within-person variability and problems with recall [102–104], that may lead to nondifferential misclassification and attenuation of the effect size in analytic studies using the exposure. In particular, this attenuation may have contributed to a lack of a significant finding from the sport domain, since the highest sport levels occurred in the distant past (prior to age 25) for most subjects, are not part of the generic memory pattern (shown to have better recall) [105] and thus may been imprecisely recalled. Despite these limitations, it has been repeatedly shown that PA questionnaires are both practical and valid when used appropriately for large-scale epidemiologic studies [86, 104, 106, 107].

The CPFI, a time-force-bodyweight product, was a stronger predictor of knee OA than any of its component parts alone and is a new measure of PA-related force measured in joint loading units. However, the CPFI does not separately and specifically capture elements of activity-related force that may be most injurious such as shear, rapid deceleration, or high-impulse loads. Activities where those elements of force occur (e.g., cutting and pivoting sports, jumping sports, and carrying heavy loads) were captured, indirectly measuring these harmful types of load, but the strength of a potential signal from these forces may have been blunted. Another potential limitation related to recall is the possibility of recall bias, where the ability to recall past exposure is dependent on outcome status. Of note, subjects with OA at baseline may have overreported prior PA exposure, attributing their OA to their past activity. This could lead to increased risk estimates, and while justified for the reasons outlined previously, the results remain vulnerable to this type of bias. However, risk estimates for sport and occupational exposure as well as other covariates were generally in the expected direction and consistent with the literature including prospective data [47–55], lending validity to the findings. Regardless, the possibility of this bias must be acknowledged, and study results interpreted in light of this.

Although this study provides evidence of an association between high levels of lifelong joint force, overweight/obesity, previous injury, BMI, and knee OA, the cross-sectional design makes the determination of a cause and effect more challenging. However, the time window used for the main PA exposure (prior to age 50) captures the ages [30–45] with the highest level of lifetime force and is separated in time from knee OA diagnosis for the vast majority of cases. Supporting this, most of the risk estimates for covariates reported in this study were in the expected direction and effect sizes consistent with the literature [47–55], and the period prevalence design included incident cases. Lastly as this study is the first attempt to examine the effect of a new exposure measure (quantitative lifelong joint load from all three primary activity domains) on knee OA, a cross-sectional approach is reasonable.

The finding that most PA-related force is not related to knee OA, but that the highest levels of joint force are, is biologically plausible and fits within the conceptual framework of causation. Under normal physiological conditions, the transmission and distribution of joint loads can occur for decades with little or no wear [108]. However, when normal joint physiologic mechanisms are overwhelmed via excessive local mechanical force, biologic events are triggered which destabilize the normal coupling of degradation and synthesis of articular cartilage and subchondral bone [109]. Animal studies clearly illustrate that high joint force from PA affects cartilage metabolism and plays a role in the development of OA [110–112].

In summary, a newly proposed measure of lifetime mechanical knee force was used to estimate the risk of self-reported knee OA. While it must be interpreted cautiously because of the cross-sectional design and the possibility of recall bias, this study suggests that lifelong physical activity is generally safe. High levels of lifetime knee force from occupational activity in men and women, and household activity in women were associated with knee OA. Obesity and previous injury were also a significant risk, consistent with previous studies. Prevention efforts may best be directed at occupations requiring high physical demands, at weight-control programs and injury prevention. Future research should further investigate the potential role of household activity, improve the estimation and validity of knee force measurement in new populations, and apply these measures in longitudinal studies. 

## Figures and Tables

**Table 1:** Subject characteristics (*n* = 4269)^∗^.

	Overall	Males	Females
*N*	4269	1575 (37%)	2694 (63%)
Mean age	61.5 (7.6)	63.0 (7.8)	60.6 (7.3)
Mean current weight (lb)	175 (41)	193 (41)	165 (38)
Mean current BMI	27.3 (5.9)	27.0 (5.3)	27.5 (6.3)
Married (%)	65.9	79.2	58.1
Some postsecondary education (%)	66	68.8	64.8

^∗^Values are the mean and SD unless otherwise indicated.

**Table 2:** Crude and adjusted^†^ odds ratios for knee OA^∗^, by sex.

	Males (*n* = 1575)			Females (*n* = 2694)		
	Prevalence (%)	Unadjusted OR (95% CI)	Adjusted OR^†^ (95% CI)	Prevalence (%)	Unadjusted OR (95% CI)	Adjusted OR^†^ (95% CI)
Knee OA^∗^	17.8			25.1		
Age						
<57	24.5	1.00	1.00	38.6	1.00	1.00
58–64	34.6	1.16 (0.82, 1.65)	1.23 (0.85, 1.79)	32.6	1.19 (0.97, 1.47)	1.25 (1.00, 1.57)
65+	40.9	1.50 (1.08, 2.09)	1.92 (1.34, 2.74)	28.0	1.40 (1.14, 1.73)	1.55 (1.23, 1.95)
Knee injury						
No	76.1	1.00	1.00	81.7	1.00	1.00
Yes	23.9	4.10 (3.16, 5.35)	4.24 (3.20, 5.62)	18.3	3.12 (2.77, 4.15)	3.17 (2.54, 3.94)
BMI^+^						
Normal	22.0	1.00	1.00	28.9	1.00	1.00
Underweight	0.8	1.21 (0.26, 5.70)	1.99 (0.40, 9.75)	1.6	1.37 (0.64, 2.93)	0.84 (0.33, 2.11)
Overweight	47.7	1.28 (0.91, 1.85)	1.09 (0.75, 1.60)	36.1	1.68 (1.32, 2.12)	1.69 (1.31, 2.19)
Obese	29.5	2.44 (1.70, 3.50)	2.20 (1.48, 3.26)	33.4	3.35 (2.65, 4.23)	3.17 (2.54, 3.94)
Total knee (TF) force						
CPFI 1	16.8	1.00	1.00	21.9	1.00	1.00
CPFI 2	21.4	1.00 (0.64, 1.56)	0.88 (0.55, 1.41)^*∧*^	19.2	1.15 (0.84, 1.54)	1.10 (0.81, 1.50)^*∧*^
CPFI 3	23.7	1.09 (0.70, 1.67)	0.95 (0.60, 1.50)^*∧*^	17.8	1.37 (1.02, 1.83)	1.27 (0.94, 1.73)^*∧*^
CPFI 4	22.0	1.49 (0.98, 2.28)	1.19 (0.72, 1.77)^*∧*^	18.8	1.42 (1.07, 1.89)	1.28 (0.95, 1.73)^*∧*^
CPFI 5	16.2	2.22 (1.44, 3.42)	1.70 (1.06, 2.70)^*∧*^	22.3	2.01 (1.53, 2.62)	1.52 (1.15, 2.02)^*∧*^

^∗^Self-reported medical diagnosis of knee OA, plus pain, aching, or stiffness most days.

^†^Adjusted for all other covariates in table.

^+^BMI categories: normal (20–24.9), underweight (<20), overweight (25–29.9), and obese (>30.0).

^*∧*^Test for trend: *P* < 0.001.

**Table 3:** Crude and adjusted^†^ odds ratios for knee OA^∗^ by activity domain and dose.

	Males (*n* = 1575)			Females (*n* = 2694)		
	Prevalence (%)	Unadjusted OR (95% CI)	Adjusted OR^†^ (95% CI)	Prevalence (%)	Unadjusted OR (95% CI)	Adjusted OR^†^ (95% CI)
Knee OA^∗^	17.8			25.1		
Sport Knee (TF) force						
CPFI.1	12.3	1.00	1.00	25.1	1.00	1.00
CPFI.2	14.7	0.84 (0.49, 1.43)	0.76 (0.40, 1.43)	23.5	0.86 (0.65, 1.13)	0.83 (0.61, 1.14)
CPFI.3	16.3	0.74 (0.43, 1.27)	0.74 (0.39, 1.39)	22.5	0.80 (0.60, 1.06)	0.72 (0.52, 0.99)
CPFI.4	23.0	1.13 (0.71, 1.82)	1.06 (0.61, 1.86)	18.0	0.81 (0.60, 1.09)	0.77 (0.55, 1.08)
CPFI.5	33.6	1.24 (0.79, 1.93)	1.13 (0.66, 1.93)	11.0	0.78 (0.55, 1.11)	0.70 (0.46, 1.05)
Occ knee (TF) force						
CPFI.1	7.5	1.00	1.00	27.6	1.00	1.00
CPFI.2	18.4	1.30 (0.68, 2.49)	1.34 (0.57, 2.61)^*∧*^	21.0	1.17 (0.89, 1.53)	1.03 (0.73, 1.43)
CPFI.3	17.3	1.60 (0.84, 3.05)	1.41 (0.56, 2.60)^*∧*^	21.7	1.25 (0.96, 1.63)	1.16 (0.84, 1.60)
CPFI.4	24.2	1.81 (0.97, 3.35)	1.62 (0.79, 3.33)^*∧*^	17.5	1.26 (0.95, 1.66)	1.05 (0.74, 1.49)
CPFI.5	32.7	2.10 (1.15, 3.82)	1.93 (0.95, 3.90)^*∧*^	12.4	1.81 (1.34, 2.44)	1.37 (0.95, 1.98)
House knee (TF) force						
CPFI.1	36.3	1.00	1.00	11.1	1.00	1.00
CPFI.2	29.9	1.01 (0.72, 1.41)	1.00 (0.68, 1.48)	14.6	0.94 (0.64, 1.38)	1.07 (0.66, 1.75)^*∧*^
CPFI.3	17.8	1.06 (0.71, 1.56)	0.94 (0.60, 1.47)	21.2	1.07 (0.75, 1.52)	1.56 (0.98, 2.43)^*∧*^
CPFI.4	9.8	0.84 (0.50, 1.40)	0.70 (0.39, 1.25)	25.6	1.24 (0.88, 1.75)	1.49 (0.97, 2.30)^*∧*^
CPFI.5	6.2	1.61 (0.94, 2.76)	1.17 (0.61, 2.24)	27.5	1.67 (1.20, 2.33)	2.02 (1.32, 3.01)^*∧*^

^∗^Self-reported medical diagnosis plus pain, aching, or stiffness most days.

^†^ORs adjusted for age, knee injury, BMI, and other activity domains.

^*∧*^Test for trend: *P* < 0.001.

**Table 4:** Sample questions from three domains of physical activity (survey was online using skip logic technology).

Purpose of questions	Specific questions	Units
Sports/recreation (using the first item in the L-PAQ sports domain, “aerobics,” as an example)		

Questions on *duration* of participation in each sport activity	Q1: At what age did you start participating in aerobics?	YOP: years of participation
	Q2: At what age did you stop participating in Aerobics? If you are still participating in Aerobics, please fill in your current age	
Questions on *frequency* of participation in each sports activity	Q3: How many months per year did you participate in Aerobics?	WPY: months per year converted to weeks per year
	Q4: How often did you participate (per week, per month, or per year)?	OPW: occasions per week (all units converted)
Questions on *length of time* of participation in one occasion of sports activity	Q5: On average, how long did you participate on each occasion (minutes or hours)?	HPO: hours per occasion (all units converted)
Questions on *hip joint movements* (e.g., time spent in a given activity—e.g., walk, stand, run/jog, squat, lift, and jump)	Q6: When participating in Aerobics, how much time did you spend doing the following activities, on average?	(ordinal radio button responses in min/hr—none, 1–5 min, <15, 15–30,…, 45–60)

Occupation (using job number 1 from L-PAQ occupational domain, as an example)		

Identify occupation	Q1: Please list job number 1	
Questions on *duration* of participation in each occupation	Q2: At what age did you start participating in job number 1?	YOP: years of participation
	Q3: At what age did you stop participating in job number 1? If you are still in job number 1, fill in your current age	
Questions on *frequency* of participation in each occupation	Q4: What type of employment was job number 1 (full time, part time, or seasonal)?	WPY: weeks per year (all units converted)
	Q5: How long was a season on average?	
Questions on *length of time* of participation in one occasion of occupation	Q6: How many hours per week did you work on average?	HPW: hours per week
Questions on *hip joint movements* (e.g., time spent in given activity—e.g., walk, stand, lift, carry, use heavy tools, squat, and lift)	Q7: When performing job number 1 how much time did you spend doing the following activities, on average?	(ordinal radio button responses in min/hr—none, 1–5 min, <15, 15–30,…, 45–60)

Household (using “caring for children” from L-PAQ household domain, as an example)		

Questions on *duration* of participation of domestic activity	Q1: At what age did you begin caring for children?	YOP: years of participation
	Q2: At what age did you stop caring for children? If you are still caring for children, fill in your current age	
Questions on *frequency* of participation of domestic activity	^ ∗^ *Assumed at 52 weeks per year*	
Questions on *length of time* performing domestic activity	Q3: How many hours per week did you care for children on average?	HPW: hours per week
Questions on *hip joint movements* (e.g., time spent in given activity—e.g., walk, stand, lift, carry, and squat)	Q4: When caring for children, how much time did you spend doing the following activities, on average?	(ordinal radio button responses in min/hr—none, 1–5 min, <15, 15–30,…, 45–60)

**Table 5:** Force value assigned to each activity in the CPFI formula. Average knee (tibiofemoral) force (× bodyweight (BW)).

Activity	Knee force (BW)
Stand	1
Walk	3
Run	6
Stand and hold object > 23 kg	1 + 23 kg
Walk and carry object > 23 kg	3 + 23 kg
Push	3
Heavy tool	1
Kneel	0
Squat	5

## Appendices

### A.

See Table 4.

### B.

See Table 5.
